# Annotated tweet data of mixed Wolof-French for detecting obnoxious messages

**DOI:** 10.1016/j.dib.2025.111500

**Published:** 2025-03-23

**Authors:** Ibrahima Ndao, Khadim Dramé, Gorgoumack Sambe, Gayo Diallo

**Affiliations:** a Computer Science and Engineering Laboratory for Innovation, Assane Seck University of Ziguinchor, Ziguinchor 27001, Senegal; b Cheikh Hamidou Kane Digital University, Dakar 10000, Senegal; c Bordeaux Population Health INSERM 1219 & LaBRI, University of Bordeaux, Bordeaux F-33000, France

**Keywords:** Abusive messages, Low-resource languages, Social networks, Natural language processing, Datasets

## Abstract

Automatic detection of obnoxious (abusive) messages on social networks is complex, especially for low-resource languages and in the case of mixed code, such as Wolof-French. This phenomenon is common in Senegalese tweets, but there is a lack of annotated data to facilitate this task. To fill this gap, we created AWOFRO, the first annotated corpus of 3510 tweets in mixed code. We analysed this corpus and validated the annotations using measures such as Cohen's Kappa.

Specifications Table

**Table:** 

Subject	Computer Sciences
Specific subject area	Collection, cleaning, and manual annotation of tweets for the detection of obnoxious (abusive) messages.
Type of data	Text (CSV file). Raw, Analyzed, Filtered, Processed.
Data collection	Tweets were collected using the Twint advanced scraping library. A dataset of 144225 tweets was extracted from January 1, 2021 to May 31, 2023 using several metadata, such as: keywords, locations or proximities, names of influential people, languages, etc. Next, 3500 tweets were selected for annotation on the basis of language mixed. The selected tweets were annotated by students speaking both languages (Wolof and French), so that each tweet was annotated by three people.
Data source location	The data was collected in Senegal. They are stored at the Engineering Computer Laboratory for Innovation, Assane Seck University of Ziguinchor, Senegal.
Data accessibility	Repository name: Zenodo Data identification number: 10.5281/zenodo.14497425 Direct URL to data: https://doi.org/10.5281/zenodo.14497425 Instructions for accessing these data: the data is open to the public
Code availability	The codes for training models are available at https://doi.org/10.17605/OSF.IO/AUD6M*.*

## 1 Value of the Data

• The dataset consists of 3500 tweets of mixed Wolof-French code. This corpus can be used to detect or identify abusive messages in social networks.
• This dataset is also very useful for researchers and organizations interested in the fields of natural language processing, code-mixing in tweets, and especially for low-resource languages such as Wolof.
• The dataset facilitates and enables governments and social media owners to have more tools for moderating digital platforms.
• The data is tagged by a group of students trained on the downstream task and understanding both languages.
• This corpus is essential both in processing the characteristics of tweets in social networks and in the code-mixing aspect.
• The dataset can also be used specifically to assess the quality of annotations and to test various forms of final annotation.

## 2 Background

Platforms like Twitter (now X) are ideal places to express opinions and sentiments about trending events [1]. This freedom of communication, while essential, leads to the dissemination of odious content, which can offend, threaten, harass, or intimidate people based on race, religion, or sexual orientation [2]. For the rest of this document, we will use the term 'abusive messages' to refer to obnoxious messages. Indeed, the substantial volume of user-generated content renders manual moderation both tedious and inefficient [3]. As a result of this, there is a pressing need for the implementation of automated solutions.

To address these challenges, automated approaches have been developed for the detection of these types of online speech [4]. However, most of the proposed methods have focused on languages with high resources such as English [5,6], Arabic [7,8], etc. Recently, a new field of research has focused on certain underserved languages such as Bengali [9,10], Eko [1], Marathi [11], or on the aspect of language mixing, such as the case of English-Hindi, for example [12,13]. The success of all these approaches lies in the availability of meticulously annotated data.

The Senegalese community is actively represented in online communications, sharing information, expressing their opinions on current affairs, etc. This was noticeable during the period from January 1, 2021 to March 31, 2023, which was marked by numerous events that eventually led to restrictions on social networks. Another distinctive feature of online Senegalese messages is the use of code mixing, for example:” Nullard rek khamoul dara quoi”. Code mixing data are texts in which two or more languages are used [12]. However, little or no research has been done on the detection of abusive messages in Senegal. To address this gap, we are actively developing the first dataset designed to identify abusive messages within Senegalese tweets.

In this paper, we introduce AWOFRO [14], the first Senegalese dataset comprising approximately 3510 mixed-code Wolof-French tweets annotated for the detection of abusive messages. This dataset is annotated using a binary annotation system, i.e. in two classes “abusive” or “non-abusive”. To manage disagreements between annotators, we ensured that exactly three annotators annotated each tweet. This approach enabled us to implement a majority voting system for the final classification. Consequently, a tweet is classified into a category if at least two annotators have assigned it to that category. We evaluate our dataset using three families of state-of-the-art models and analyze the outcomes in detail. The data is freely available to the research community.

## 3 Data Description

The creation of an annotated corpus requires tedious work. Rigorous work and considerable time-consuming effort are essential, particularly for local languages such as Wolof [15]. Non-compliance with spelling and grammatical rules and the use of mixed codes (Wolof-French) make this task even more complex. To our knowledge, there are no annotated corpora available for the detection of abusive messages of mixed code Wolof-French data. We are therefore endeavouring to produce the first ever dataset for the detection of abusive messages in a mixed code Wolof-French context.

### 3.1 Data Collection

Collecting data on platforms such as Twitter (now X) requires appropriate tools. For this, we used the Twint tool. This is an advanced Python library for collecting data on Twitter. We chose this tool because of its ease of use and its ability to retrieve tweets without having to use the Twitter API (which imposes a limit of 3200 tweets). Metadata includes tweets from specific users, tweets related to a specific word or to certain topics or to a well-defined date, hashtags. Several queries were formulated using:

• A set of keywords: World Cup, racist, politician, insulting terms, etc.
• A set of places name or proximity names: Senegal, Dakar, Qatar, Cameroon, Morocco, Ghana, Algeria, Tunisia, Africa, etc.
• Names of potential targets: Aliou Cisse, Macky Sall, Ousmane Sonko, etc.

These queries allow for covering various cases and contexts of abusive messages publication. We were able to collect 144,225 tweets during the period from 1 January 2021 to 31 May 2023. This period was particularly marked by the many sociopolitical events in Senegal that led to Internet restrictions. The raw data collected contains both monolingual and multilingual tweets. In order to resolve ethical issues and preserve confidentiality, we have removed all metadata and personally identifiable information. The collected data are in a tabular format.

### 3.2 Data Annotation

The annotation process involved several steps that are described below.

#### 3.2.1 Pre-Processing

In this step, a great deal of manual work was performed to select tweets in mixed code (Wolof-French) from the corpus. In addition to this selection, emojis, URLs, mentions, and hashtags were removed in order to reduce the noise and focus on the code-mixing aspect studied here.

#### 3.2.2 Preparation of the Annotation Protocol

This step includes the preparation of the annotation tool, the selection and training of the annotators. The annotations were carried out using Google forms and Google apps script. A script was developed to create a form in which the questions are the tweets to be annotated and the answers are radio buttons with two options: ‘abusive’ or ‘not abusive’. A single submission authorization has been configured to prevent a given user from completing the form several times. We selected native speakers who have a good understanding of Wolof and French. Although the downstream task is subjective, it has been demonstrated in the literature that annotations provided by trained annotators yield much better results. However, we trained all our annotators in the task and the annotation protocol. The annotation took place in two phases: (i) a first phase with 18 undergraduate students and (ii) a second phase with 15 postgraduate computer science students.

Abusive language can be subtle and difficult to detect, even in humans. To ensure accuracy in our study, we provided annotators with a clear definition of abusive language. We instructed them to consider a message abusive if it:

• Targets individuals or groups: Attacks or excessively criticizes people based on characteristics like ethnicity, religion, or sexual orientation.
• Causes offense: Is offensive to individuals or groups based on characteristics like ethnicity, religion, or sexual orientation.

This definition, adapted from [16], aims to capture both explicit and implicit forms of abuse.

#### 3.2.3 Annotation Phase

To ensure accuracy and consistency in our annotation process, we implemented the following methodology:

• Individual Annotation: Annotators were assigned to groups of three, but each annotator worked independently to avoid bias and ensure individual judgment.
• Standardized Units: Tweets were grouped into sets of 100 and distributed to three annotators per set.
• Binary Classification: Each tweet was classified into one of two categories: ``abusive'' or ``not-abusive.''
• Data Consolidation: Annotations were collected and compiled into a central Tabular file for analysis.

**Fig. 1:**
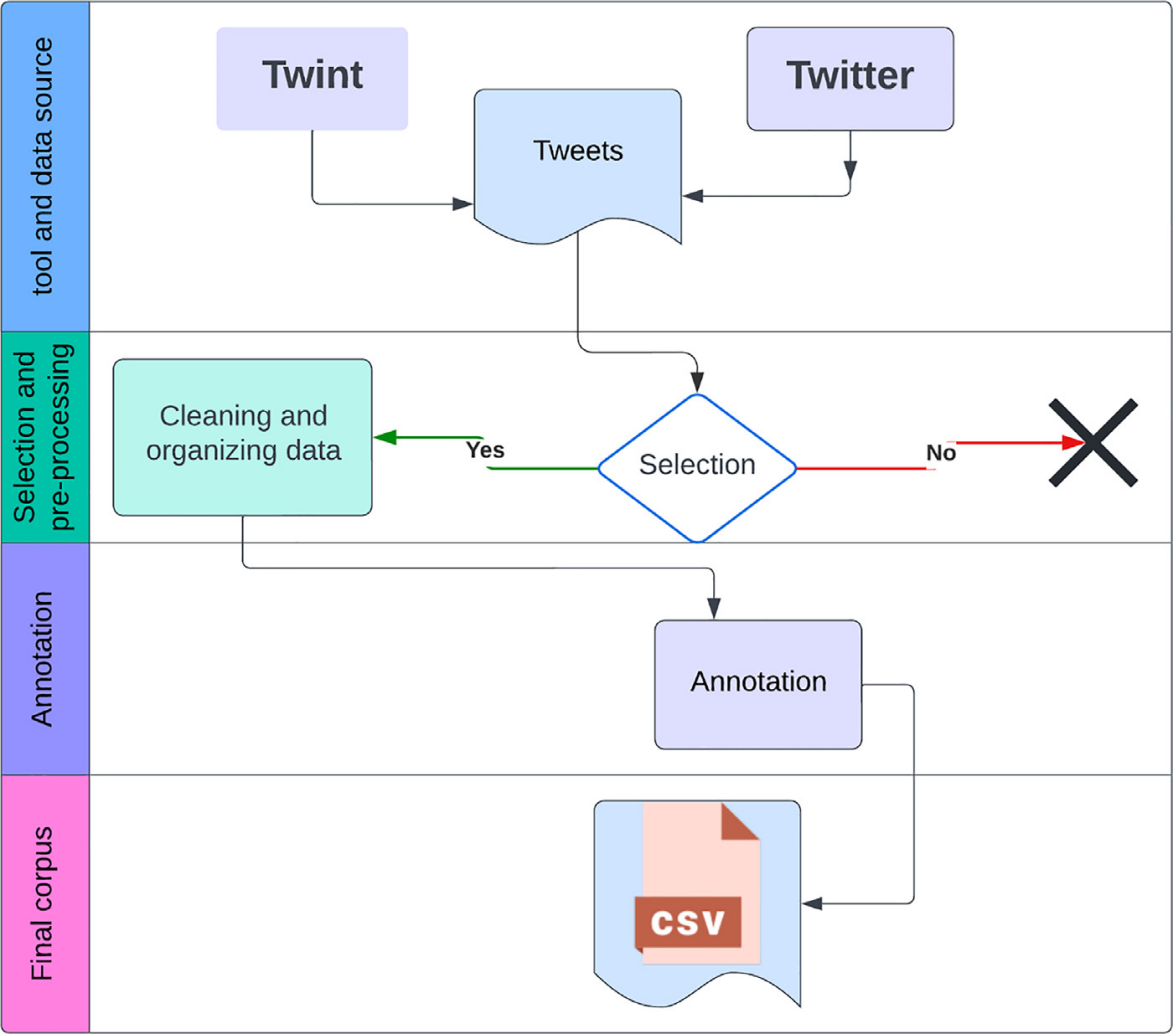
Collection, preprocessing and annotation processes.

In total, 3510 tweets were annotated, with each tweet receiving three independent assessments. This rigorous process, illustrated in Fig. 1, aimed to maximize the reliability of our data.

In order to obtain a definitive annotation for each tweet and manage the divergence between annotators, we used a majority vote. Thus, a tweet is definitively annotated in a class if at least two of the annotators have categorized it in that class. Finally, the files were merged into a csv file with several columns such as the tweets column, the annotations of the three annotators (Annot1, Annot2, Annot3), the number of annotations as “abusive” (CountA), the number of annotations as “not abusive” (CountNA) and the majority vote (Class). Table 1 shows a visual of the annotated corpus data in the Tweets column.

**Table 1:** Examples of messages from the annotated corpus.

Tweets	English translation	Annot1	Annot2	Annot3	CountNA	CountA	Class
Waaw coupons la wala coupures	but are they coupons or are they cuts	Non abusi	Non abusif	Abusif	2	1	0
Sory kaba merde fen-rek guay deff ragualal yalla	Sory kaba shit you're just lying fear god	Abusif	Abusif	Abusif	0	3	1
yakamte doul rek le cas assane devait lui servir de lecon	hasten to lie the assane case should serve as a lesson to him	Abusif	Abusif	Non abusive	1	2	1

### 3.3 Datasets Statistics

**Fig. 2:**
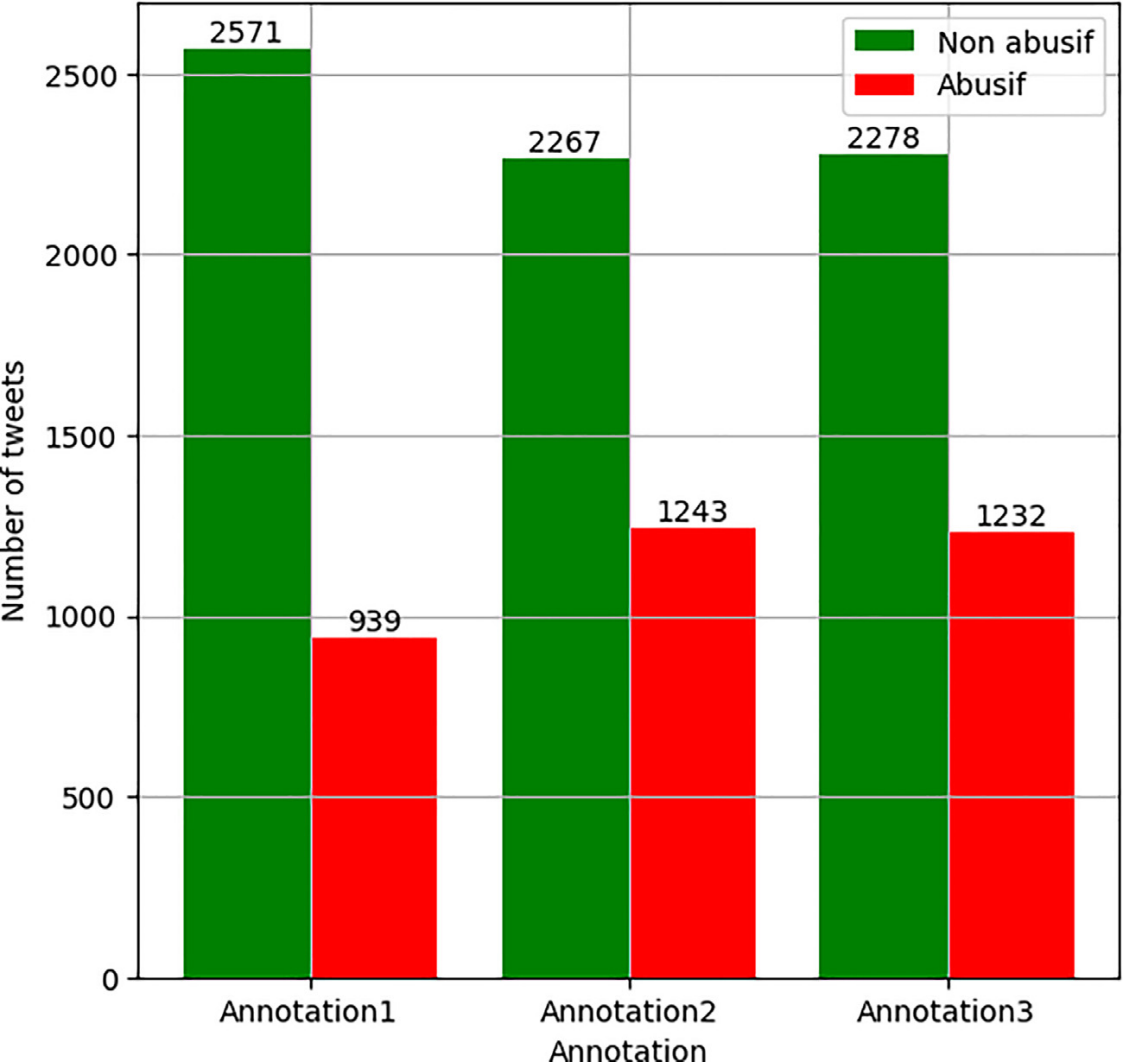
Breakdown of abusive and not-abusive annotations for each annotator.

As described in the previous sections, AWOFRO consists of 3510 annotated tweets. The collection of tweets includes keywords, names of places, and influential people. A preliminary pre-processing was carried out by selecting tweets with the Wolof-French code mix and removing certain metadata deemed irrelevant for the code mix aspect studied here. We then opted for a binary annotation for the tags: abusive or not-abusive. Fig. 2 shows the distribution of labels (abusive and not-abusive) in relation to the three annotations.

**Fig. 3:**
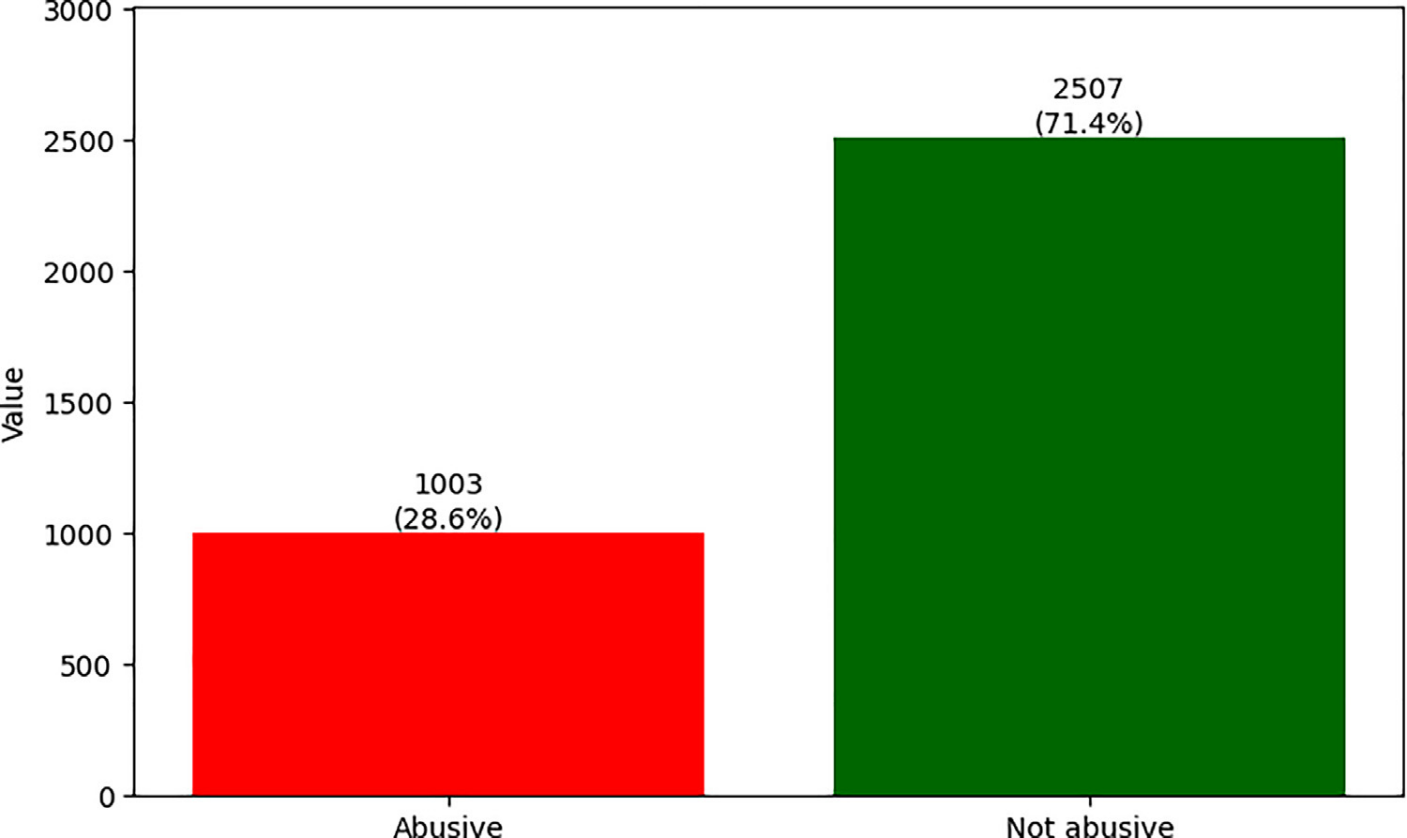
The ratio of annotations after the majority vote.

A majority vote was used as inter-annotator agreement. In other words, a tweet T is definitively classified in class C1 if it has been classified in C1 by at least two of the three annotators. Thus, the corpus of 3510 tweets is divided into 2510 tweets belonging to the not-abusive class, i.e. 71.51 %, and 1000 tweets annotated as abusive, i.e. 28.49 %. Fig. 3 shows the ratio of annotations after the majority vote.

This report confirms that the number of not-abusive tweets on social networks far exceeds the number of abusive tweets. Nevertheless, the volume of abusive tweets and the negative impact they can have demands considerable attention. At the same time, the problem of imbalance between classes is a common remark in existing corpora on the detection of abusive messages. In addition, the task of detecting abusive messages, known for its subjectivity, deserves rigorous and conscientious work when annotating the data. In our corpus, we can note that out of the 2507 tweets annotated as ‘not abusive’, the three annotators agreed on the 1525 tweets, i.e. an agreement of 60.83 %. For the ‘abusive’ category, on the 1003 tweets annotated as ‘abusive’, the annotators agreed on 426 tweets, i.e. 42.47 %. This demonstrates once again the complexity of this task, where even humans struggle to reach perfect agreement, particularly in the ‘abusive’ category [17]. What's more, automating this task can be more difficult. The Table 2 shows the ratio of agreement between annotators.

**Table 2:** Inter-annotator agreement report.

number of annotators in agreement	Not abusive	Abusive
3 annotators	1525	426
2 annotators	982	577

To guarantee the validity, reliability and consistency of our annotations provided, we use inter-annotator agreement measures. These inter-annotator agreement measures are used to estimate the biases in the annotation of such a corpus. This helps to analyse the quality and robustness of the annotations and to evaluate the results produced by the models.

Various measures are used in the literature to assess the reliability of annotations. They include the Cohen's Kappa and the Fleiss’ Kappa coefficients.

#### 3.3.1 Cohen's Kappa

The Cohen's Kappa test is a measure for assigning an agreement value between -1 and 1 between two annotators on qualitative data [18]. This statistical measure is one of the most widely used because of its reliability in studies where reproducibility is paramount. Cohen's Kappa coefficient measures the agreement between two qualitative annotations [19]. This coefficient is the sum of a random proportion and a real proportion. It is defined by Formula (1):

(1) K=(Po−Pe)/(1−Pe)

with, Po: the actual proportion of agreement

Pe: the proportion of random agreement

The closer the coefficient K is to 1, the more reliable the agreement between annotators. The case where Po = Pe (coefficient K=0) shows that there is no relationship between the annotations (the resulting agreements or disagreements are considered to be the result of chance). On the other hand, when the value of K is equal to -1, we have a total disagreement.

The Table 3 shows the interpretation of Cohen's Kappa K coefficient.

**Table 3:** Interpretation of Cohen's Kappa K coefficient [19].

Interpretation	coefficient K
Excellent	> 0.81
Good	0.80 - 0.61
Moderate	0.60 - 0.21
Poor	0.20 - 0.0
Very Poor	< 0.0

#### 3.3.2 Fleiss’ Kappa

The Fleiss’ Kappa coefficient builds on the Cohen's Kappa coefficient calculation to evaluate annotations from more than two annotators. Given Ai annotations, i(1,..,n), the Fleiss’ Kappa coefficient is the ratio of the weighted sums of the Ai [20].

#### 3.3.3 Krippendorff's Alpha

Krippendorff's α is a measure of the annotation reliability of several annotators on categorical, ordinal or nominal data [21]. Krippendorff's α is between the values 0 and 1, where if α =0 corresponds to total disagreement and α =1 corresponds to perfect agreement. It is calculated by the Formula (2):

(2) α=1−Do/De

with, Do: the disagreement derived from the evaluated data

De: the disagreement expected at random

The Table 4 shows the interpretation of the values of Krippendorff's α

**Table 4:** Interpretation of Krippendorff's α values.

Interpretation	coefficient α
Full agreement	1
Satisfactory	0.80–0.91
Moderate	0.68–0.79
Low	0.1–0.67
Full disagreement	0

#### 3.3.4 Comparison Between the Kappa Coefficient and the Alpha Coefficient

The Table 5 shows a comparative study of the Kappa K coefficient and Krippendorff's α coefficient.

**Table 5:** Comparative table of the Kappa K coefficient and Krippendorff's Alpha coefficient.

Option	Kappa coefficient K	Krippendorff's α coefficient
Taking chance into account	Yes	Applicabilité
Applicability	Categorical, Nominal, ordinal	Categorical, Nominal, ordinal
Category dependency	Yes	Yes
Dependence on corpus size	No	Yes
Complexity of calculation	No	Yes
Easily interpreted	Yes	No

Based on the above comparison, we used the Kappa coefficient (Cohen and Fleiss) to assess the reliability and consistency between annotations.

We evaluated the annotations in two stages:

• In the first stage, we used Cohen's Kappa coefficient to evaluate annotations in pairs (A1 vs A2, A1 vs A3 and A2 vs A3).
• In the second stage, the Fleiss Kappa coefficient is used to evaluate the three annotations.

Cohen's Kappa coefficient measurements gave 0.32, 0.40 and 0.35 respectively for the A1 vs A2, A1 vs A3 and A2 vs A3 agreements.

The Fleiss Kappa coefficient for the three annotations was 0.36. From the interpretations of the K coefficient defined in the Table 3 suggest, we can conclude that our pairwise or global annotations are satisfactory (moderate). Given the subjective nature of our research domain, a moderate coefficient of agreement is acceptable for research reproducibility [20].

## 4 Experimental Design, Materials and Methods

In this section, we present some experiments demonstrating the quality and validity of AWOFRO. However, we have chosen models that have demonstrated a certain performance in previous work. Thus, three families of models are trained, evaluated and presented here. We present the performance of three machine learning models: Support Vector Machine (SVM), Naive Bayes (NB) and Logistic Regression (LR); three deep learning models: Convolutional Neural Networks (CNN), Long Short-Term Memory (LSTM) and Gated Recurrent Unit (GRU); and three language models (bert-base-multilingual-cased, camembert-base, Davlan/bert-base-multilingual-cased-finetuned-wolof) on the downstream task. To this end, we divided our dataset into 80 % for training and 20 % for testing. We use class-based evaluation to analyse the ability of the models to identify different classes. We used the scikit learn library to develop machine learning models with word2vec text embeddings, known for their effectiveness in capturing semantic information across data. For the deep learning models, the TensorFlow library was used. The first layers of the LSTM and GRU models use a 100-dimensional vector representation of words. These representations are passed to the next layer of 128 neurons to capture long-term information before being passed to the dense (output layer) with a single output. A sigmoid function is applied to obtain binary outputs (either 0 or 1). The CNN model, on the other hand, uses a one-dimensional convolutional layer with 128 channels of size 5 each with a Relu activation function to convert negative values to 0 for better data convergence. The result will be fed to a pooling layer to merge the results of the previous layers into a feature vector, and then routed to the last layer (output layer) with the Sigmoid activation function for binary classification. For all the deep learning models, we used the binary_crossentropy loss function to minimise the loss during training; we also used the Adam parameter optimiser to update the model weights. Each model was trained over 10 epochs and a batch_size of 32. For the language models (pretrained models), we used the transformers library and PyTorch to load and re-train the models on the downstream task. All three models presented here are available on the Hugging Face platform. The hyperparameters used for training are: a maximum length of input sequences during training is set to 128, a batch size is set to 16, a learning rate of 2e-5 to optimise weights and training over 5 epochs.

The Table 6 below shows the results obtained after evaluating the models described above. The best results per class are highlighted in bold.

**Table 6:** Evaluation of models.

Category	Models	Precision		Rappel		F-mesure		Accuracy
		0	1	0	1	0	1	
ML Models	SVM	**0.76**	0.55	0.84	**0.41**	0.80	**0.47**	0.71
	LR	0.74	0.59	0.91	0.30	0.81	0.40	**0.72**
	NB	0.73	**0.65**	**0.94**	0.26	**0.82**	0.37	**0.72**
DL Models	CNN	0.74	0.58	0.90	0.31	0.81	0.40	0.71
	LSTM	0.72	0.49	0.87	0.28	0.79	0.35	0.68
	GRU	0.73	0.49	0.84	0.34	0.78	0.40	0.68
Language Models	bert-base-multilingual-cased	0.74	0.60	0.91	0.29	**0.82**	0.39	**0.72**
	camembert-base	0.73	0.52	0.88	0.29	0.80	0.37	0.69
	davlan/bert-base- multilingual-cased- finetuned-wolof	0.72	0.60	0.93	0.22	0.81	0.32	0.71

These different experiments provide clear insights into the usefulness of the dataset. These models were chosen based on their proven performance in previous studies. However, our experiments show that the SVM model obtained the best accuracy on class “0” with 0.76, while the Naïve Bayes (NB) model has the best accuracy for class “1” with 0.65. The NB with 0.94 and the SVM with 0.41 for class “0” and “1” respectively obtain the best recalls. The Bert-base-multilingual-cased model and the Naïve Bayes obtain the best F-measure for class “0” with 0.82 and the SVM model for class “1” with 0.47. However, the Logistic Regression (LR), Naïve Bayes and BERT models slightly outperformed the others in terms of accuracy with 0.72.

The evaluations of our different models reveal poorer performance for class “1”, which can be explained by the low representativeness of class “1” on the dataset. In addition, language models and deep learning models require a fairly large amount of data.

## Limitations

The subjectivity of detecting abusive messages makes it almost impossible to produce a data set that can include all linguistic and cultural diversities. In addition, the different classes existing in the data annotations of abusive messages make our dataset generalizable or even comparable to other datasets.

In addition to careful selection and training of annotators, we recognize that the use of students can inevitably include additional biases to the task. However, we share the various annotations with researchers who are interested in this issue.

Data imbalance is a common problem with almost all data sets on the detection of abusive messages. We argue that the latter could have clearly impacted our models because we did not use over-sampling, under-sampling or class weighting.

## Ethics Statement

The data presented in this paper was obtained using Twitter's services strictly adhering to their Terms of Service. As per Twitter's copyright policy, tweets may be owned by various entities. However, we have safeguarded the privacy rights of individuals in this dataset by removing each user's identity from the tweets and comments.

## CRediT authorship contribution statement

**Ibrahima Ndao:** Writing – original draft, Methodology, Software, Validation. **Khadim Dramé:** Conceptualization, Resources, Writing – review & editing, Supervision. **Gorgoumack Sambe:** Resources, Writing – review & editing, Supervision. **Gayo Diallo:** Conceptualization, Writing – review & editing, Supervision.

## Data Availability

(Zenodo).AWOFRO : Annotated tweet corpus of mixed Wolof-French for detecting obnoxious messages (Original data)
